# Levels of Oxidized LDL, Estrogens, and Progesterone in Placenta Tissues and Serum Paraoxonase Activity in Preeclampsia

**DOI:** 10.1155/2013/862982

**Published:** 2013-03-28

**Authors:** Şerefden Açıkgöz, Ülkü Özmen Bayar, Murat Can, Berrak Güven, Görkem Mungan, Suat Doğan, Vildan Sümbüloğlu

**Affiliations:** ^1^Department of Biochemistry, Faculty of Medicine, Bülent Ecevit University, 67630 Zonguldak, Turkey; ^2^Department of Gynecology and Obstetrics, Faculty of Medicine, Bülent Ecevit University, 67630 Zonguldak, Turkey; ^3^Department of Biostatistics, Faculty of Medicine, Bülent Ecevit University, 67630 Zonguldak, Turkey

## Abstract

In vitro literature studies have suggested that atherosclerotic oxidized low density lipoprotein (OxLDL) inhibits trophoblast invasion. The objective of this study was to determine the levels of OxLDL and to examine the relationship between antioxidative estradiol, estriol, and prooxidative progestin in normal and preeclamptic placental tissues and measure the serum activity of antioxidative paraoxonase (PON1). The study included 30 preeclamptic and 32 normal pregnant women. OxLDL was determined with ELISA, estradiol, unconjugated estriol, and progesterone that were determined with chemiluminescence method in placental tissues. Serum PON1 activity was determined with spectrophotometric method. Levels of OxLDL (*P* = 0.027), estriol (*P* < 0.001), estradiol (*P* = 0.008), and progesterone (*P* = 0.009) were lower in the placental tissues of preeclamptic group compared to the normal pregnant women. Serum PON1 activity was higher in preeclamptic group (*P* = 0.040) and preeclamptic group without intrauterine growth restriction (*P* = 0.008) compared to normal pregnant women. Tissue estriol of preeclamptic group without/with IUGR (*P* < 0.001, *P* = 0.002) was lower than the normal group. Results of our study suggest that the events leading to fetoplacental insufficiency lead to a reduction in the levels of estriol limit deposition of OxLDL in placental tissues. The serum PON1 activity is probably important in the inhibition of OxLDL in preeclampsia.

## 1. Introduction

Preeclampsia is a syndrome characterized by hypertension and proteinuria leading to maternal and perinatal mortality [1]. The etiopathogenesis of preeclampsia has not been fully understood to date. It is believed that in preeclampsia, the physiological remodeling of the uterine spiral arteries into dilated uteroplacental vessels observed in normal pregnancies is disrupted [2].

Indeed, medial hyperplasia and atherosis of the spiral arteries have been reported [2]. Acute atherosis is an occlusive lesion of the spiral arteries with an unknown time of onset characterized with infiltration by foam cells that store cytoplasmic fat [3]. Oxidation of low density lipoproteins (LDL) to oxidized LDL (OxLDL) is a triggering and accelerating factor in the development of atherosclerotic lesions [4]. Oxidative stress is one of the stimulating factors taking part in the inflammatory response of pregnancy [3]. 

The placenta is the major source of prooxidant agents, antioxidant enzyme systems and hormones, and is able to keep the lipid peroxidation under control in normal pregnancy. The balance between prooxidative and antioxidative metabolites affects the lag phase of LDL oxidation [5]. 

Estradiol inhibits LDL oxidation, whereas progesterone increases LDL oxidation in primary tissue cultures of term human placenta [6]. PON1 is an antioxidative enzyme found in the high density lipoprotein and acts as protective against in vitro oxidative modification of LDL [7]. 

Since spiral artery disease due to atherosis is suggested to be involved in the pathogenesis of preeclampsia, it seemed reasonable to examine levels of OxLDL because of its atherogenic role.

Our review of the literature failed to find a report that has done OxLDL measurements directly in placental tissues in preeclampsia. We hypothesized that atherogenic OxLDL might be increased in preeclamptic placental tissues. The objective of our study was to measure the levels of OxLDL and investigate the relationship between OxLDL and prooxidant progestin and antioxidant estrogens in preeclamptic placental tissues and placental tissues of normal pregnant women. Furthermore, PON1 activity was investigated in preeclamptic and normal serum because of its antioxidative property. 

## 2. Material and Methods

The patients in this study were all Turkish women. Thirty women with preeclampsia and 32 women with normotensive, uncomplicated pregnancies who delivered at the Department of Obstetrics and Gynecology, Bülent Ecevit University, were included in the study after obtaining approval from the Faculty Ethics Committee. Written informed consent was obtained from each patient prior to the performance of any study procedures.

The diagnosis of preeclampsia was established in accordance with the definition of the American College of Obstetricians and Gynecologist [8]. 

After delivery, fetal intrauterine growth restriction (IUGR) was defined as birthweight <10th percentile as assessed by gestational age at delivery with the definition of Alexander et al. [9].

Samples were taken from the central region of the placenta and placed on ice, transported to the laboratory, processed within 30 min, rinsed in ice cold PBS, and immediately frozen at –80°C for analysis at a later date. Tissue samples were minced and homogenized at 1/1 rate with phosphate buffer saline at pH 7.4 using a homogenizer (Ultra Turrax IKA T18 Basic). The homogenates were centrifuged at 9000 ×g at 4°C for 30 minutes and the supernatants were immediately analyzed. The levels of OxLDL, estradiol, unconjugated estriol, and progesterone were measured in supernatant, following centrifugation, and the results were calculated as per gram tissue. Blood samples were obtained prior to the onset of labor. After the blood clotted, the samples were centrifuged and aliquots of serum were immediately stored at −80°C until assayed for PON1 activity. All analyses completed on blinded samples and they ran in duplicate.

Levels of oxLDL were determined using the Human OxLDL ELISA kit (Cusabio Biotech Catalog no. CSB-E07931 h, China) with the sandwich method in EPSON LX-300 ELISA device (BIO-TEC Instruments, Winooski, USA) with an intra-assay CV of <3% and interassay CV of <10%. ELISA kit was specific for total OxLDL. 

Levels of estradiol (intra-assay CV of <4.9%, interassay CV of <7.1%), unconjugated estriol (intra-assay CV of <5.2%, interassay CV of <7.3%), and progesterone (intra-assay CV of <7.0%, interassay CV of <9.5%) were assayed using an Immulite 2000 device (Siemens, CA, USA), with the solid phase, competitive chemiluminescent enzyme immunoassay.

PON1 activity was measured using Shimadzu UV 1601 spectrophotometer (Shimadzu Co., Kyoto, Japan) according to the definition of Gan et al. with and intra-assay CV of <8.8%. One mMol of paraoxon was used as substrate in the presence of 1 mMol of CaCl_2_ in 100 mMol Tris-HCl buffer (pH 8.0); serum was added to start the reaction, and the increase in absorbance at 412 nm was recorded. The amount of p-nitrophenol was calculated from the molar extinction coefficient (17.100 M^-1 ^cm^−1^). Results were expressed as U/L [10]. 

## 3. Statistical Analysis

The statistical package for social sciences 13.0 (SPSS Inc, Chicago, IL, USA) was used in all data analyses. The Kolmogorov-Smirnov test was used to test the normality of distributions. Student *t*-test was used to test the significance of differences between preeclamptic and normal study groups with normal distribution. 

Three group comparisons of the variables with normal distribution were done with one way analysis of variance (ANOVA). Bonferroni test was used as a post-hoc test for significant variables. For the variables without normal distribution, Kruskal-Wallis test was used for three group comparisons and Mann Whitney *U*-test with Bonferroni adjustment used for two group comparisons of significant variables. Pearson's and Spearman's correlation analyses were used to determine the relationship between continuous variables state measurement.

Power calculations for the related comparison tests were done with PASS 2008 package programme. *P* values less than 0.05 were considered statistically significant.

## 4. Results

Demographic (maternal age, BMI), clinical (systolic and diastolic blood pressures, urinary protein excretion per day, infant's birth weight, scores of Apgar at 1 and 5 minutes) and biochemical characteristics (tissue and serum), and correlation coefficients of tissue markers and clinical data are shown in Tables 1, 2, 3, and 4, respectively. Biochemical characteristics of normal pregnant women (group 1), preeclamptic pregnant women without IUGR (group 2), and preeclamptic pregnant women with IUGR (group 3) are shown in Tables 5 and 6. 

Mean gestational weeks (GW) were 35.1 ± 4.4 in the preeclamptic group and 37.4 ± 1.5 in the control group. The rates of vaginal delivery/C-section were 9/23 in normal group, 2/28 in preeclamptic patients. Nulliparity/multiparity were 18/14 in normal group and 18/12 in preeclamptic group. Pregnant women in both groups had quit smoking throughout their pregnancies. 

The OxLDL (*P* = 0.027), estriol (*P* < 0.001), estradiol (*P* = 0.008), and progesterone (*P* = 0.009) levels of the placental tissues in preeclamptic patients were significantly lower than in the healthy pregnant women using student *t*-test (*P* < 0.05). After Bonferroni adjustment, tissue estriol, estradiol, and progesterone levels were found to be statistically significant in the preeclamptic patients compared to those of the normal pregnant women, whereas the levels of OxLDL were not different significantly (Table 2). 

Serum PON1 activity (*P* = 0.040) was significantly higher in the preeclamptic group compared to that of the normal pregnant women (Table 3). 

A positive correlation between the tissue OxLDL-tissue estriol levels (*r* = 0.36, *P* = 0.004) and between tissue estriol-infant's birth weight (*r* = 0.39, *P* = 0.002) was given in Table 4. A negative correlation between the tissue estriol levels and systolic BP (*r* = −0.44, *P* < 0.001) and diastolic BP (*r* = −0.48, *P* < 0.001) was given in Table 4. 

The estriol (*P* < 0.001), estradiol (*P* = 0.007), and progesterone (*P* = 0.007) levels of the placental tissues and serum PON1 (*P* = 0.025) were different significantly between normal pregnant group, preeclamptic group without IUGR, preeclamptic group with IUGR (Tables 5 and 6). 

Comparison between the groups is shown in Tables 5 and 6. 

Power calculation of the biochemical data revealed 62.6% for OxLDL, 99% for estriol, 79% for estradiol, 76.9% for progesterone, and 54.4% for PON 1.

## 5. Discussion

Several pathological processes occurring in the preeclamptic placenta are thought to be involved in the pathogenesis, including impaired spiral artery remodeling [2], endothelial dysfunction, impaired placental trophoblastic implantation, and inadequate perfusion of uterine-placental unit [11]. Atherosclerotic endothelial alterations triggered by oxidative stress have also been stressed in the pathogenesis [1]. Oxidation of LDL secondary to oxidative stress and transformation of monocytes into foam cells following the uptake of OxLDL are significant steps towards endothelial dysfunction [11].

### 5.1. OxLDL

Pavan et al. have demonstrated the presence of OxLDL in villous and extravillous cytotrophoblasts of human placenta in the first trimester using immunohistochemistry, and reported in a cell culture study that OxLDL leads to defective trophoblast invasion [12].

There is no consensus on the levels of OxLDL in preeclamptic women in the literature. Kim et al. [13] and Genç et al. [14] have reported that serum OxLDL levels were higher in the preeclamptic pregnant women compared to the normal pregnant women, whereas others Pecks et al. [15] and Raıjmakers et al. [16] have reported lower levels. 

Our results are in conjunction with those of Pecks et al. and Raıjmakers et al. In our study, differing from the previous studies, we examined the levels of OxLDL in placental tissues.

Raıjmakers et al. have determined lower OxLDL levels in the serum of preeclamptic pregnant women compared to the controls and suggested that autoantibodies against OxLDL could accelerate the serum clearance of OxLDL [16].

Pecks et al. did not find increased levels of serum OxLDL in preeclamptic patients contrary to their hypothesis. They found lower serum OxLDL levels in preeclampsia and significantly lower serum OxLDL levels in IUGR (normotensive IUGR+IUGR with preeclampsia) patients compared to normals. Their measurements were done in serum samples; although they could not demonstrate a systemic alteration, they underlined the importance of probable factors (local effects) within the placenta tissues and maternal vessels. They speculated the diminished fetal cholesterol supply as a probable mechanism responsible from the low OxLDL levels [15]. In our study, we failed to demonstrate increased levels of OxLDL within the placental tissues. 

In our study, there were not significant differences between the levels of OxLDL in the preeclamptic pregnant women with IUGR in comparison with normal pregnant women and preeclamptic patients without IUGR. 

### 5.2. Steroid Hormones

Several studies in the literature have demonstrated the presence of OxLDL in placenta [12] and the effects of antioxidant estrogens and prooxidant progesterone on LDL oxidation [5, 6, 17, 18]. These sex hormones are synthesized in placental tissue during gestation [5], and cholesterol is the precursor of both. Muraguchi et al. have reported that estradiol indicates placental function, whereas estriol indicates placental and fetal function [19]. Zhu et al. have reported that progestin inhibits the antioxidant effect of estradiol in the absence and presence of aortic endothelial cell culture [17, 18] and that this effect is similar to that observed in trophoblastic cell cultures [6]. Mueller et al. have reported that estriol is an antioxidant that increases the lag phase of LDL oxidation in vitro [5]. 

In our study, levels of estriol, estradiol, and progesterone were reduced in the placental tissues of preeclamptic patients compared to normal pregnant women. Those gave the impression that the in vivo reduction in OxLDL in preeclamptic placenta tissues had a different mechanism from the antioxidant effect of estrogens.

Estriol was significantly reduced in the placental tissues of preeclamptic patients and preeclamptic patients without/with IUGR compared to normal pregnancy group. This implied significancy of estriol in evaluating placental and fetal growth as reported by Muraguchi et al. [19]. 

We suggest that the reduction in steroid hormones synthesized from cholesterol in preeclamptic placental tissues might be associated with the reduction in fetal cholesterol maintenance as reported by Pecks et al. [15].

### 5.3. PON1

Genç et al. [14], Sarandöl et al. [20], Baker et al. [21], and Yaghmaei et al. [22] have measured PON1 activity using the same method as in our study (hydrolysis of paraoxon by PON1). 

Yaghmaei et al. have determined that serum PON1 activity was significantly increased in preeclampsia compared to the normal subjects [22].

Baker et al. have determined higher paraoxonase activity in preeclamptic pregnant women compared to controls. Authors have also reported higher paraoxonase activity in severe compared to mild preeclampsia and stressed the importance of paraoxonase in the pathogenesis of preeclampsia [21].

Genc has reported lower PON1 in preeclamptic pregnant women compared to the controls [14].

Sarandöl et al. have measured paraoxonase activity in normal, mild, and severe preeclamptic pregnant women. Authors have reported no differences between the groups [20]. 

The results of our study were compatible with those of Yaghmaei and Baker and suggested that elevated serum PON1 activity in preeclampsia might be important in the in vivo inhibition of OxLDL. PON1 activity of preeclamptic pregnant women without IUGR was significantly higher than the normal group. It might be important in the progression of fetus.

Mackness et al. investigated PON 1 and PON2 in several tissues by real-time polymerase chain reaction (PCR) and showed PON 2 in placenta tissues, but they stated that the wide tissue distribution of PON1 would theoretically lead to greater protection for oxidative stress [23]. In preeclampsia regarding our results, the potential role of PON 1 activity on fetal growth can be explained by further studies.

Our literature search failed to find a report that has done OxLDL measurements directly in placental tissues. We hypothesized that atherogenic OxLDL might be increased in preeclamptic placental tissues and aimed to demonstrate the relationship with sex steroids in placental tissue. However, our results demonstrated that levels of OxLDL and steroid hormones were reduced in placental tissues in preeclampsia. Therefore, we believe that reductions in tissue estriol and OxLDL are correlated, and this positive correlation, although not a strong one, might in turn be associated with a reduction in the maintenance of fetal cholesterol. Additionally, these reductions in placental tissues might also be associated with the rapid removal of the possible complexes formed by OxLDL and autoantibodies by RES cells. However, we were unable to demonstrate the increase of OxLDL in preeclamptic placental tissues. Further studies should be performed to investigate the role of other factors acting in spiral artery atherosis and multifactorial endothelial dysfunction. 

## Figures and Tables

**Table 1:** Demographic and clinical data of the study subjects.

	Normal pregnant women (*n* = 32)	Preeclamptic pregnant women (*n* = 30)	*P* value
Mean ± SD			

Maternal age, years	27 ± 6	30 ± 6	0.078
Gestational age, weeks	37.4 ± 1.5	35.1 ± 4.4	**0.009**
Infant's birth weight, g	3172 ± 369	2429 ± 907	**0.001**
BMI	27.6 ± 1.5	28.6 ± 1.1	**0.008**

Median (min-max)			

Systolic BP, mm Hg	110 (90–140)	150 (140–210)	**0.001**
Diastolic BP, mm Hg	70 (50–100)	90 (90–120)	**0.001**
Urine mg protein/day	37.6 (7.90–200)	921 (307–6923)	**0.001**
Apgar 1 min	9 (3–10)	9 (0–10)	**0.038**
Apgar 5 min	10 (5–10)	10 (0–10)	**0.023**

Statistically significant *P* < 0.05.

**Table 2:** Levels of OxLDL, estriol, estradiol, and progesterone in the placenta of preeclamptic and normal pregnant women.

Placental tissue	Normal pregnant women (*n* = 32)	Preeclamptic pregnant women (*n* = 30)	*P* value
OxLDL, *µ*Mol/mL/g tissue	9.85 ± 6.82	6.54 ± 4.42	0.027
Estriol, ng/mL/g tissue	67.6 ± 23.1	41.9 ± 23.2	**0.001**
Estradiol, pg/mL/g tissue	25.9 ± 18.0	14.6 ± 14.2	** 0.008**
Progesterone, ng/mL/g tissue	0.34 ± 0.31	0.16 ± 0.21	** 0.009**

Mean ± SD

Statistically significant *P* < 0.01.

**Table 3:** Serum PON1 activity in preeclamptic and normal pregnant women.

Serum	Normal pregnant women (*n* = 32)	Preeclamptic pregnant women (*n* = 30)	*P* value
PON1, U/L	121 ± 80	174 ± 115	**0.040**

Mean ± SD

Statistically significant *P* < 0.05.

**Table 4:** Correlation coefficients of tissue markers and clinical data (*n* = 62).

	Correlation coefficient (*r*)	*P* value
Tissue estriol-tissue OxLDL	0.36	** 0.004**
Tissue estriol-GW	0.25	>0.05
Tissue OxLDL-GW	0.04	>0.05
Tissue estriol-infant's birth weight	0.39	** 0.002**
Tissue estriol-systolic BP	−0.44	**0.001**
Tissue estriol-diastolic BP	−0.48	**0.001**

Statistically significant *P* < 0.05.

**Table 5:** Levels of OxLDL, estriol, estradiol, and progesterone in the placenta of preeclamptic without/with and normal pregnant women.

Placental tissue	Normal pregnant women (*n* = 32) group 1	Preeclamptic pregnant women without IUGR (*n* = 21) group 2	Preeclamptic pregnant women with IUGR (*n* = 9) group 3	*P* value
Mean ± SD				

OxLDL, *µ*Mol/mL/g tissue	9.85 ± 6.82	5.88 ± 4.41	8.09 ± 4.26	0.058
Estriol, ng/mL/g tissue	67.6 ± 23.1	43.5 ± 24.6*	38.4 ± 20.6*	**0.001**

Median (min-max)				

Estradiol, pg/mL/g tissue	20.3 (6.2–62.5)	8.2 (5.6–37.2)*	11.4 (5.3–61.3)	**0.007 **
Progesterone, ng/mL/g tissue	0.22 (0.06–1.18)	0.08 (0.06–0.45)*	0.11 (0.05–0.97)	**0.007**

Statistically significant *P* < 0.05

*Differ from the normal group: (group 1-2: estriol *P* < 0.001, group 1–3: estriol *P* = 0.002, group 1-2: estradiol *P* = 0.001, group 1-2: progesterone *P* = 0.001).

**Table 6:** Serum PON1 activity in preeclamptic without/with and normal pregnant women.

Serum	Normal pregnant women (*n* = 32) group 1	Preeclamptic pregnant women without IUGR (*n* = 21) group 2	Preeclamptic pregnant women with IUGR (*n* = 9) group 3	*P* value
PON1, U/L	116 (22–288)	185 (50–520)*	86 (70–213)	**0.025**

Median (Min–Max)

Statistically significant *P* < 0.05

*Differ from the normal group: group 1-2: PON1 *P* = 0.008.
